# Extracellular vesicles from dHL-60 cells as delivery vehicles for diverse therapeutics

**DOI:** 10.1038/s41598-021-87891-8

**Published:** 2021-04-15

**Authors:** Jun-Kyu Kim, Young-Jin Youn, Yu-Bin Lee, Sun-Hwa Kim, Dong-Keun Song, Minsang Shin, Hee Kyung Jin, Jae-sung Bae, Sanjeeb Shrestha, Chang-Won Hong

**Affiliations:** 1 Department of Physiology, School of Medicine, Kyungpook National University, 680 Gukchaebosang-ro, Daegu, 41944 Republic of Korea; 2 Department of Pharmacology, College of Medicine, Hallym University, Chuncheon, 24252 Republic of Korea; 3 Department of Microbiology, School of Medicine, Kyungpook National University, Daegu, 41944 Republic of Korea; 4 Department of Laboratory Animal Medicine, College of Veterinary Medicine, Kyungpook National University, Daegu, 41944 Republic of Korea; 5 KNU Alzheimer’s Disease Research Institute, Kyungpook National University, Daegu, 41566 Republic of Korea

**Keywords:** Neutrophils, Drug delivery

## Abstract

Extracellular vesicles (EVs) are membrane-derived heterogeneous vesicles that mediate intercellular communications. They have recently been considered as ideal vehicles for drug-delivery systems, and immune cells are suggested as a potential source for drug-loaded EVs. In this study, we investigated the possibility of neutrophils as a source for drug-loaded EVs. Neutrophil-like differentiated human promyelocytic leukemia cells (dHL-60) produced massive amounts of EVs within 1 h. The dHL-60 cells are also easily loaded with various cargoes such as antibiotics (penicillin), anticancer drug (paclitaxel), chemoattractant (MCP-1), miRNA, and Cas9. The EVs derived from the dHL-60 cells showed efficient incorporation of these cargoes and significant effector functions, such as bactericidal activity, monocyte chemotaxis, and macrophage polarization. Our results suggest that neutrophils or neutrophil-like promyelocytic cells could be an attractive source for drug-delivery EVs.

## Introduction

Extracellular vesicles (EVs) are membrane-derived heterogeneous vesicles that are enclosed by lipid bilayers^1^. Almost all kinds of living cells generate EVs, ranging in size from nanometers (exosomes) to micrometers (apoptotic bodies) meters^1,2^. The EVs mediate intercellular communications by transferring packaged biological information, such as proteins, lipids, and nucleic acids^1–6^. Recently, EV is considered to be an ideal target for a drug-delivery vehicle because of its unique characteristics, such as long systemic circulation time, low degradation rate, low systemic toxicity, robust cargo preservation, and easy absorption by target cells^7–9^. Therefore, they have been utilized to deliver of therapeutic small-molecule drugs for specific diseases, tissue repair, and regeneration^7–10^. However, limitations remain for developing EVs as drug-delivering vehicles. Therefore, many studies are trying to develop a method to shorten the time required for EV generation, force cells to generate EVs, and enhance the specificity of EVs against target cells. Recently, studies have focused on developing of drug-delivery using EVs derived from immune cells to avoid possible harmful effects to host^11–14^.

Neutrophils are highly mobile immune cells recruited to the inflammatory site to fight against invading pathogens. They are equipped with various receptors and ligands that enable them to interact with a specific target cell. Neutrophils also generate EVs during the inflammatory process. Neutrophil-derived EVs (NDEVs) are classified into three major groups upon the site of generation: exosomes, microvesicles, and trails^15^. NDEVs contain various molecules, such as RNA, proteins, and lipids. They actively participate in intercellular communications and modulation of disease conditions^15^. Activated neutrophils release tremendous amounts of EVs than cancer cells or nonimmune cells^15^, within few minutes after external stimulation. Moreover, NDEVs are equipped with various ligands and receptors that target pathogens and other immune cells. Therefore, NDEVs could be an attractive vehicle for developing a drug-delivery system targeting specific pathogens or immune cells. However, the short life span of neutrophils has limited the development of a drug-delivery system using NDEVs. HL-60 cells, promyelocytic leukemia cells, are widely used as a model system to study neutrophil function^16,17^. Neutrophil-like cells are easily differentiated from HL-60 cells. These neutrophil-like differentiated HL-60 (dHL-60) cells have neutrophil characteristics, such as reactive oxygen species (ROS) generation, neutrophil extracellular trap (NET) formation, chemotaxis, and bactericidal activity^17–20^. Moreover, they have a short-life span due to spontaneous apoptosis^21^ which is an advantage over the EVs generated from general cancer cells. Therefore, recent studies employed dHL-60 cells to investigate NDEVs^22,23^ and found the beneficial effects on inflammatory diseases, such as acute lung injury and stroke^22,23^.

Here, we investigated the incorporation of various cargoes in dHL-60 cell-derived EVs. The dHL-60 cells produce large amounts of EVs within a short time and EVs derived from the dHL-60 cells showed similar characteristics to NDEVs. In addition, the dHL-60 cell-derived EVs were successfully loaded with various cargoes, such as antibiotics, anti-cancer drugs, and Cas-9, and these cargo-loaded EVs showed efficient therapeutic effects according to the types of cargo.

## Materials and methods

### Differentiation of HL-60 cells

Human myeloblastic cells (HL-60 cells) were cultured and maintained in RPMI 1640 (Gibco) supplemented with 5% heat-inactivated fetal bovine serum (Hyclone) and 1 × antibiotic/antimycotic (Gibco) at 37 °C and 5% CO_2_. Human myeloblastic cells (HL-60 cells), Jukart cells, and THP-1 cells were cultured and maintained in RPMI 1640 (Gibco) supplemented with 10% heat-inactivated fetal bovine serum (Hyclone) and 1 × antibiotic/antimycotic (Gibco) at 37 °C and 5% CO_2_. The MCF-7 and COLO205 cells were cultured and maintained in RPMI 1640 (Gibco) supplemented with 10% heat-inactivated fetal bovine serum (Hyclone) and 1 × antibiotic/antimycotic (Gibco) at 37 °C and 5% CO_2_. For the differentiation of neutrophil-like differentiated HL-60 cells, 1 × 10^5^ cells/ml were treated with 1 μM all-transretionic acid (ATRA, Sigma-Aldrich). The cultures were washed and reseeded in fresh media followed by ATRA treatment every third day up to the sixth day. For differentiation of THP-1, 2 × 10^6^ cells were treated with phorbol 12-myristate 13-acetate (PMA, Sigma-Aldrich, 50 ng) for 48 h.

### Live-imaging of EVs formation

Naïve HL-60 cells or dHL-60 cells (10^6^ cells) were stained with calcein-AM (2 μg/ml, Merk Millipore) and seeded in a confocal dish (SPL Life Sciences) pre-coated with 0.1% poly-L-lysine (Sigma-Aldrich). The cells were visualized for EVs formation by fluorescence microscopy (Olympus IX83, Olympus) in the presence or absence of 1 μM fMLP (Sigma-Aldrich).

### Isolation of EVs

EVs were isolated from supernatants collected after the culture of naïve HL-60 or dHL-60 cells in the presence or absence of fMLP. The supernatants were collected, and the EVs were purified by centrifugation at 2500 rpm, followed by filtration through a 1.2 μm filter (Ministart Syringe Filter). The filtered EVs containing supernatants were ultracentrifuged at 100,000 × g for 1 h at 4 °C. The pelleted EVs were resuspended into 1 × PBS and stored at − 70 °C.

### The quantification of EVs

The EVs were isolated from naïve HL-60, dHL-60, or HEK293 cells prestained with calcein-AM (5 μg/ml). The fluorescence of calcein was measured using a spectrophotometer (Spectromax M2/e fluorescence microplate reader, Molecular devices).

### Nanoparticle-tracking analysis (NTA)

The isolated EVs were resuspended into 1 × PBS and further diluted. NTA analysis was performed using a Nanosight LM10 nanoparticle characterization system (Malvern Instruments). The NTA analytical software version 3 was used for capturing and analyzing data.

### Scanning electron microscopic analysis

The EVs were absorbed on the surface of the filter disc, incubated in 3% glutaraldehyde (Sigma-Aldrich), dehydrated using ethanol (Merk Millipore), dried with liquid CO_2_, and sputtered with osmium. Samples were visualized using a Hitachi SU8229 scanning electron microscope (Hitachi).

### The separation of NDEVs

Human blood experiment was approved by the Institutional Research Board of Kyungpook National University and proceeded according to the institutional guidelines. Venous blood was taken from healthy adult volunteers (age ≥ 18). All participants provided informed consent following the Declaration of Helsinki. Neutrophils were purified using density gradient as described previously^24^. Isolated neutrophils (5 × 10^6^ cells) were stimulated with either fMLP (1 μM) or *S. aureus* (1 × 10^6^ cells) for 20 min. *S. aureus* (10^9^ cells) were opsonized for 30 min with autologous serum (100 μl). The supernatants were collected, and EVs were isolated as previously described^25^. In brief, remnant cells and debris were removed and filtered through a 1.2 μm filter (Minisart Syringe Filter, Sartorius) and further concentrated using ultracentrifugation at 100,000 × *g* for 60 min. The concentrated EVs were dissolved in 100 μl RPMI. The supernatants were exposed to penicillin–streptomycin (Pen-Strep, Sigma-Aldrich) for 30 min before centrifugation to eliminate remnant bacteria in NDEVs.

### Cargo loading in EVs

Either naïve HL-60 or dHL-60 cells were transfected with various cargoes (i.e., penicillin, Sigma-Aldrich; paclitaxel, Invitrogen; monocyte chemoattractant protein-1, MCP-1, Sinobiologicals; miR-16 mimic, Abm; miR-16 precursor, Ambion; Cas-9-GFP, provided by Prof. Minsang-Shin) using NEPA21 electroporator (NEPA gene) at 225 V. To validate the transfection efficacy, the cargoes were conjugated with various fluorescent: BOCILLIN-FL penicillin, Invitrogen; oregon green conjugated paclitaxel, Flutax-2, Invitrogen; Atto 488 MCP-1, Atto 488 labeling kit, Sigma-Aldrich; miRNA, labeled using ULYSIS Nucleic acid labeling kit, Molecular Probes; Cas9-GFP. The transfected cells were analyzed using a flow cytometer to determine the fluorescence levels of each cargo. For comparison, the HEK293 cells were transfected with cargoes using a NEPA21 electroporator and the EVs were isolated.

### Bactericidal activity of EVs

*S. aureus* (ATCC 25,923) was opsonized with human serum for 30 min at 37 °C. Serum was obtained from clotted blood by centrifugation. The opsonized *S. aureus* (1 × 10^6^ cells/ml) were exposed to the EVs (10^8^ particles) for 30 min at 37 °C. Bacteria were then spread into an LB agar (Difco) and colony-forming units were counted following incubation for 18 h at 37 °C. Bacteria were exposed to EVs (10^8^ particles) in the presence of annexin A5 (AnxA5) or lysed EVs to inhibit the effector function of penicillin-loaded EVs. The EVs were lysed with SDS following a previous study^26^. The EVs were lysed with 0.025% SDS, vortexed for 1 min, centrifuged at 14,000*g* for 10 min, and the supernatants were removed. The pellets were washed twice with ice-chilled 1 × PBS and resuspended.

### Chemotaxis of monocytes against EVs

Monocytes were isolated from the peripheral blood mononuclear cells layer obtained after histopaque centrifugation and purified using a percoll solution as described previously^27^. The chemotaxis of monocytes against the EVs was determined using a μ-slide chamber (ibidi) following the manufacturer’s instructions. One side of the chemotaxis chamber was loaded with the EVs, and monocyte were allowed to migrate. For comparison, one side of the chemotaxis chamber was loaded with MCP-1, and monocyte chemotaxis was assessed. For the inhibition of monocyte chemotaxis against EVs, monocytes were pretreated with CCR2 inhibitor (1 μg/ml, Santacruz) for 30 min and allowed to migrate toward EVs in the presence of the CCR2 inhibitor. The cell movements were visualized and captured by the Nikon Eclipse Ni-U microscope using × 20 objective. The chemotaxis was then analyzed using ImageJ and chemotaxis/migration tool (ibidi).

### Tumoricidal effects of drug-loaded EVs

MCF-7 (Korean Cell Line Bank) and COLO205 (Korean Cell Line Bank) were exposed to either vehicle or paclitaxel-loaded EVs for 24 h. The viability of cancer cells was assayed using the MTT (3-[4,5-dimethylthiazol-2-yl]-2,5 diphenyltetrazolium bromide) assay kit (Invitrogen) following the manufacturer’s instructions.

### Polarization of differentiated THP-1

THP-1 cells were purchased from Korean Cell Line Bank. THP-1 cells were maintained at 5 × 10^6^ cells/ml in RPMI (Gibco) supplemented with 10% heat-inactivated FBS (HyClone, GE healthcare Life Sciences) with 1% penicillin/streptomycin (Sigma-Aldrich). THP-1 cells were differentiated into M0 macrophages by treatment with PMA (100 ng/ml) for 48 h. M0-differentiated THP-1 cells (10^6^ cells) to EVs. For comparison, M0-differentiated THP-1 cells were stimulated with either LPS (100 ng/ml) or IFN-γ (10 ng/ml) for M1 polarization or IL-4 (10 ng/ml) and recombinant human IL-12 (10 ng/ml, Sinobiologicals) for M2 polarization. RNA was extracted from polarized-THP-1 cells using TRIzol (Ambion), and cDNA was synthesized using the RT^2^ First strand kit according to manufacturer’s recommended protocols (Qiagen). RT-qPCR was performed by using the cDNA (100 ng), primer (1 μl), and RT^2^ SYBR Green qPCR mastermix (Qiagen) using Qiagen rotor. Predesigned RT^2^ qPCR primer assays (Qiagen) were used to determine the expression level of iNOS (NOS2, PPH00173F) and Arginase-1 (PPH20977A). Gene expression levels were calculated as log_2_(2^−ΔΔct^) using GAPDH (PPH00150F) as a control. For some experiments, M0-differentiated THP-1 cells were exposed to the EVs in presence of AnxA5 or lysed EVs.

### The loading of Cas9 in EVs

MCF7, Jukart (Korean Cell Line Bank), and differentiated THP-1 (THP-1, Korean Cell Line Bank) were exposed to either vehicle or Cas9-loaded EVs for 2 h at 37 °C. The cells were collected and fixed. A flow cytometer examined the expression of Cas9-GFP in the harvested cells.

### Statistical analysis

Data are presented as the mean ± SEM for continuous variables and as the number (%) for the categorical variables. Data are compared using the Student's t-test (unpaired, two-sided) and a p value of less than 0.05 was considered statistically significant. The statistical data were analyzed by Graphpad prism 7.0 (GraphPad Software Inc).

## Results

### The dHL-60 release a heterogeneous population of EVs

The HL-60 cells were allowed to differentiate into neutrophil-like dHL-60 cells by treatment with all-trans-retinoic acid (ATRA). The ATRA-treated HL-60 cells showed increased segmentation of nuclear lobes (Supplementary Fig. 1a) without significant changes in apoptosis (Supplementary Fig. 1b). dHL-60 cells also showed increased expressions of CD11b, CD18, and CD54 with decreased expressions of CD15 and CD33 (Supplementary Fig. 2a). These results are in accordance with previous studies that reported surface marker expressions of myeloid leukemic cells toward neutrophils^28,29^. We further confirmed the effector functions of the dHL-60 cells. The dHL-60 cells successfully generated reactive oxygen species (ROS) in response to PMA stimulation and showed spontaneous NET formation (Supplementary Fig. 2b,c) but did not enhance the expression of markers for granules (Supplementary Fig. 2d).

**Figure 1:**
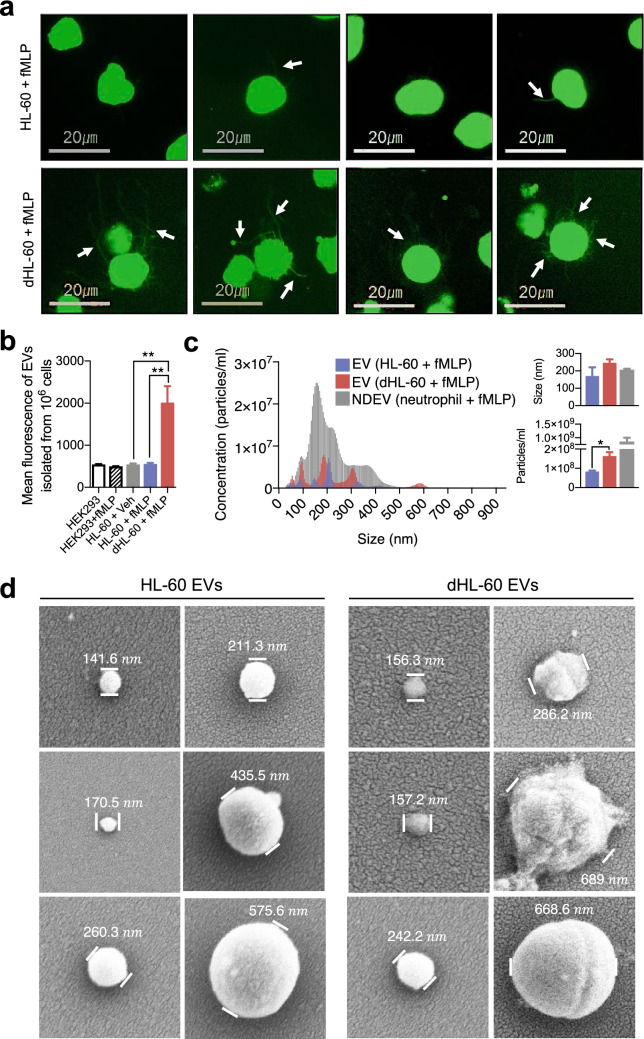
The naïve HL-60 cells and dHL-60 cells release a heterogeneous population of EVs. (**a**) Representative images showing EVs released from HL-60 cells and dHL-60 cells stimulated with fMLP. The arrow indicates membrane protrusions during EVs release. (**b**) Quantification of relative amounts of EVs derived from the naïve HL-60 and dHL-60 cells pre-stained with calcein-AM in the presence or absence of fMLP stimulation. (**c**) A representative plot showing the size and concentration of EVs derived from the HL-60 cells, dHL-60 cells, and neutrophils stimulated with fMLP. (**d**) Scanning electron microscopic analysis of EVs generated from HL-60 and dHL-60 cells. The data shown are the mean ± SEM. *P < 0.05; **P < 0.01.

Next, we examined whether dHL-60 cells generate EVs. The spontaneous generation of EVs from cells is a prolonged process. Hence, various methods are developed to force cells to generate EVs^30^. We employed a biological approach using N-formyl-methionyl-leucyl- phenylalanine (fMLP), a bacterial-derived protein, because neutrophils generate EVs in response to fMLP stimulation^31–33^. The naïve HL-60 and dHL-60 cells were loaded with calcein-AM, stimulated with fMLP for 90 min, and live-cell fluorescence images were obtained using fluorescence microscopy. The dHL-60 cells showed a significantly enhanced production of EVs compared to those from the naïve HL-60 cells (Fig. 1a, upper panel). They showed numerous protrusions that extended from membranes (Fig. 1a, arrows), which were finally detached from cell membranes. The EVs derived from the naïve HL-60 cells and dHL-60 cells were isolated, and fluorescence was measured using a spectrofluorometer. The dHL-60 cells produced significantly large amounts of EVs in response to fMLP stimulation compared to those from the naïve HL-60 or HEK293 cells (Fig. 1b). The nanoparticle tracking analysis (NTA) showed that the EVs generated from the naïve HL-60 and dHL-60 cells are heterogenous in size ranging from 50 to 600 nm in diameter (mean diameter of 170.5 ± 49.4 nm versus 246.8 ± 19.5 nm) compared to the NDEVs that were isolated from fMLP-stimulated neutrophils (Fig. 1c). We further examined the morphology of the EVs derived from the naïve HL-60 and dHL-60 cells using electron microscopy. Scanning electron microscopic (SEM) analysis identified that both EVs have an oval-shaped morphology with heterogeneous diameters (Fig. 1d). Next, we evaluated the functions of EVs isolated from the naïve HL-60 cells and dHL-60 cells. Both EVs did not show bactericidal activity (Supplementary Fig. 3a) and did not induce chemotaxis of monocytes (Supplementary Fig. 3b).

These results suggest that dHL-60 cells generate massive amounts of EVs that are phenotypically similar to NDEVs with negligible effector functions. Therefore, we thought they could be an attractive vehicle for drug delivery and investigated their possible therapeutic use on drug-delivery systems based on the dHL-60-derived EVs.

### The delivery of anti-bacterial and anti-cancer drugs using the dHL-60 derived EVs

**Figure 2:**
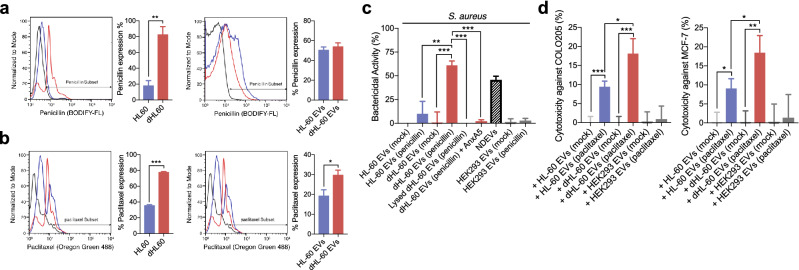
The delivery of penicillin and paclitaxel using dHL-60 cell-derived EVs. (**a**) Left panels, fluorescence levels of penicillin in the HL-60 and dHL-60 cells. Right panels, fluorescence levels of penicillin in the EVs derived from the HL-60 and dHL-60 cells. (**b**) Left panels, fluorescence levels of paclitaxel in the HL-60 and dHL-60 cells. Right panels, fluorescence levels of paclitaxel in EVs derived from the HL-60 and dHL-60 cells. (**c**) Bactericidal activity of penicillin-loaded EVs against *S. aureus.* (**d**) Cytotoxicity of paclitaxel-loaded EVs against COLO205 and MCF-7. All data represent three independent experiments; mean ± SEM. *p < 0.05; **P < 0.01; ***P < 0.001.

Recent studies showed that antibiotics and anticancer drugs are efficiently derived through EVs^13,34^. Therefore, we evaluated the delivery of these drugs through the dHL-60 cell-derived EVs. The naïve HL-60 and dHL-60 cells were loaded with fluorescence-tagged penicillin and paclitaxel using an electroporator. Flowcytometric analysis showed that both drugs were successfully incorporated into parent cells (Left panels in Fig. 2a,b). The EVs derived from both cells showed significant amounts of fluorescence from drugs derived from the parent cells (Right panels in Fig. 2a,b). The dHL-60 cells showed higher efficacy of incorporating both drugs, and only paclitaxel showed significantly higher incorporation in the dHL-60 cell-derived EVs.

We further confirmed the effectiveness of these drug-loaded EVs. The bactericidal activity of the drug-loaded EVs was examined. The NDEVs isolated from neutrophils stimulated with *S. aureus* showed significant bactericidal activity as previously reported^25^. Interestingly, the penicillin-loaded EVs derived from the dHL-60 cells showed significant bactericidal activity against *S. aureus*, whereas the penicillin-loaded EVs derived from the HL-60 cells did not (Fig. 2c). The EVs derived from HEK293 cells did not show bactericidal activity against *S. aureus* (Fig. 2c). We further confirmed the effects of lysed EVs on the bactericidal activity of the penicillin-loaded EVs. The lysed EVs did not show bactericidal activity against *S. aureus* (Fig. 2c). We further inhibited the bactericidal activity of the EVs derived from the penicillin-loaded dHL-60 cells using annexin A5 (AnxA5), an inhibitor for phosphatidylserine expressed on the EV membrane. The AnxA5 completely inhibited the bactericidal activity of the EVs derived from the penicillin-loaded dHL-60 cells (Fig. 2c).

We further examined the anticancer effects of the drug-loaded EVs. The anti-cancer effect of the drug-loaded EVs was compared to the EVs isolated from the paclitaxel-transfected HEK293 cells. Both paclitaxel-loaded EVs showed significant anticancer effects on COLO205 and MCF-7 (Fig. 2d). However, the paclitaxel-loaded EVs derived from the dHL-60 cells showed higher cytotoxic effects against both cancer cells compared to those from HL-60 cells and paclitaxel-loaded HEK293 cells (Fig. 2d). Collectively, these results suggest that the EVs derived from the dHL-60 cells could be an effective vehicle for antibiotics and anti-cancer drugs.

### Delivery of MCP-1 using the dHL-60 derived microvesicles

**Figure 3:**
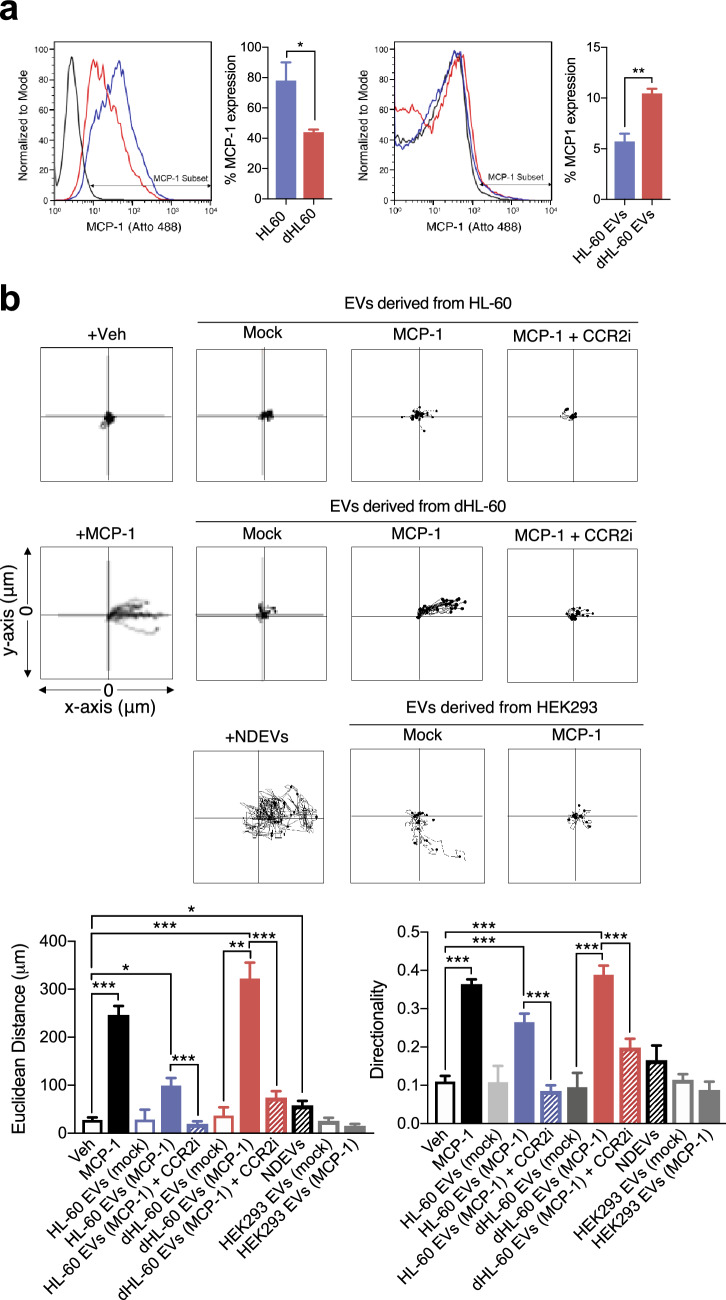
The delivery of MCP-1 using dHL-60 cell-derived EVs. (**a**) Left panels, fluorescence levels of MCP-1 in the HL-60 and dHL-60 cells. Right panels, fluorescence levels of MCP-1 in EVs derived from the HL-60 and dHL-60 cells. (**b**) Monocyte migration tracking analysis against EVs. Bar graphs denote Euclidean distance and relative directionality. All data represent three independent experiments; mean ± SEM. *P < 0.05; **P < 0.01; ***P < 0.001.

Next, we evaluated whether diffusible molecules such as chemotactic proteins can be delivered through EVs. We transfected fluorescence-tagged monocyte chemoattractant protein-1 (MCP-1, 1 mg/ml) in the naïve HL-60 or dHL-60 cells using an electroporator. Flowcytometric analysis showed that MCP-1 was effectively incorporated in both cells (Fig. 3a, left panel) and EVs were derived from both cells (Fig. 3a, right panel). Although transfection efficacy of the HL-60 cells with MCP-1 was significantly higher than that of the dHL60 cells, dHL-60 cell-derived EVs showed higher rates of MCP-1 incorporation.

To confirm the successful incorporation of MCP-1 in EVs, we examined the effects of MCP-1 incorporated EVs on monocyte chemotaxis. Both MCP-1 loaded EVs effectively induced chemotaxis of monocytes with increased directionality and distance coverage (Fig. 3b). Moreover, monocyte showed a slightly higher rate of chemotaxis against EVs isolated from the dHL-60 cells loaded with MCP-1 than against either an equal concentration of MCP-1 or NDEVs (Fig. 3b). EVs isolated from HEK293 cells loaded MCP-1 did not induce chemotaxis of monocytes (Fig. 3b).

We further examined the effect of the pharmacological inhibition of C–C chemokine receptor 2 (CCR2), a receptor for MCP-1, on monocyte chemotaxis against MCP-1-loaded EVs. The CCR2 antagonist (CCR2i) completely inhibited monocyte chemotaxis against MCP-1-loaded EVs (Fig. 3b). These results suggest that a diffusible chemoattractant can be successfully incorporated into dHL-60 cell-derived EVs.

### The delivery of micro-RNA using the dHL-60 derived microvesicles

**Figure 4:**
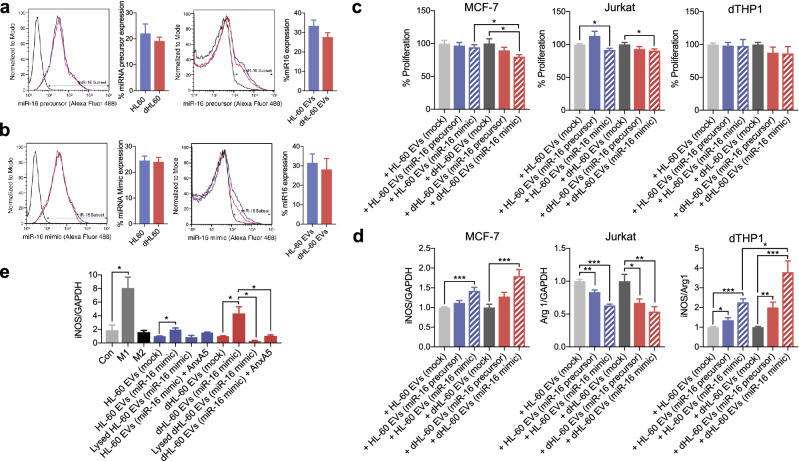
The delivery of miRNA-16 using dHL-60 cell-derived EVs. (**a**) Left panels, fluorescence levels of the miRNA-16 precursor in the HL-60 and dHL-60 cells. Right panels, fluorescence levels of the miRNA-16 precursor in EVs derived from the HL-60 and dHL-60 cells. (**b**) Left panels, fluorescence levels of the miRNA-16 mimic in the HL-60 and dHL-60 cells. Right panels, fluorescence levels of the miRNA-16 mimic in EVs derived from the HL-60 and dHL-60 cells. (**c**) The effects of EVs on the proliferation of the MCF-7, Jurkat, and dTHP-1 cells. (**d**) The effects of EVs on expressions of iNOS and arginase in MCF-7, Jurkat, and dTHP-1 cells. (**e**) The expression of iNOS in the dTHP-1 cells exposed to the HL-60 and dHL-60 cells. All data represent three independent experiments; mean ± SEM. *P < 0.05; **P < 0.01; ***P < 0.001.

MicroRNAs (miRNAs) are small non-coding RNA molecules that mediate the post-translational regulation of gene expressions^35,36^. To examine the possibility of EVs as a miRNA-delivery vehicle, we examined the incorporation of miRNAs in the dHL-60 cell-derived EVs. The naïve HL-60 and dHL-60 cells were transfected with fluorescence-labeled synthetic miR-16 (5 nM). The miR-16 is known to induce apoptosis of tumor cells, induce polarization of macrophages into the M1 phenotype, and activate CD4^+^ T cells by downregulation of PD-L1^37,38^. The miR-16 (precursor and mimic) were successfully incorporated into both cells and loaded in both EVs (Fig. 4a,b). The miR-16 loaded EVs significantly inhibited the proliferation of MCF-7 and Jurkat cells but not the THP-1-derived macrophages (dTHP1) (Fig. 4c). However, miR-16 loaded EVs significantly enhanced the expression of iNOS and attenuated the expression of arginase (Fig. 4d). We further confirmed the uptake of the dHL-60 derived EVs by the target cells. EVs were isolated from the dHL-60 cells transfected with miR-16 and M0-differentiated THP-1 cells were exposed to EVs loaded with miR-16. Then, RNA was extracted from the THP-1 cells, cDNA was synthesized, and subsequently used as a template for qPCR using the miR-16 primer. The THP-1 cells exposed to EVs derived from the dHL-60 cells loaded with miR-16 showed significantly high levels of miR-16 (Supplementary Fig. 4d). We finally confirmed the requirement of interaction between dHL-60 derived EVs and target cells. The expression of iNOS in EV-exposed THP-1 cells was completely inhibited by the EV lysis, anti-AnxA1 antibody, and AnxA5 (Fig. 4e). These results suggest that EVs derived from HL-60 cells and dHL-60 cells could be utilized to deliver miRNAs with functional modalities.

### The delivery of Cas9-RNP using the dHL-60 derived microvesicles

**Figure 5:**
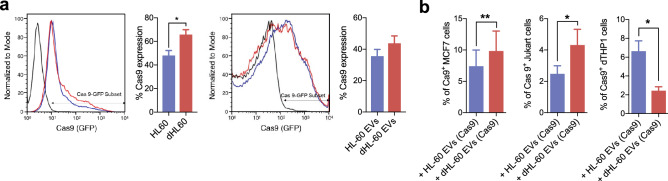
The delivery of Cas 9-GFP using dHL-60 cell-derived EVs. (**a**) Left panels, fluorescence levels of Cas9 in the HL-60 and dHL-60 cells. Right panels, fluorescence levels of Cas9 in EVs derived from the HL-60 and dHL-60 cells. (**b**) Fluorescence levels of Cas9 in the MCF-7, Jurkat, and dTHP-1 cells exposed to EVs. All data represent three independent experiments; mean ± SEM. *P < 0.05; **P < 0.01.

Although clustered regularly interspaced short palindromic repeats (CRISPR)/CRISPR-associated protein-9 nuclease (Cas9) provides robust genome editing, efficient methods for delivering them into target cells are still required. To date, electroporation is the primary choice for delivering CRISPR/Cas9 to target cells^39,40^. We examined whether EVs derived from the HL-60 cells could be efficient vehicles for delivering Cas9. The naïve HL-60 and the dHL-60 cells were transfected with GFP-tagged Cas9. The Cas9-GFP were successfully incorporated in both cells, and EVs were derived from both cells (Fig. 5a). Next, we examined the delivery of Cas9-GFP-loaded EVs against target cells. The dTHP-1 cells, MCF-7 cells, and Jurkat cells were exposed to the Cas9-GFP-loaded EVs, and the GFP fluorescence levels were measured. The MCF-7 cells showed the highest GFP fluorescence, and the Jurkat cells showed negligible GFP fluorescence levels (Fig. 5b). These results suggest that the dHL-60 cell-derived EVs could be candidate vehicles for CRISPR/Cas9.

## Discussion

In this study, we found that the HL-60 and dHL-60 cells generated EVs in response to external stimulation. These EVs were successfully loaded with various cargoes and showed functional modalities. However, the dHL-60 cells produced significantly larger amounts of EVs than HL-60 cells, and most cargoes were more efficiently loaded in EVs derived from the dHL-60 cells. Moreover, the cargoes-loaded EVs derived from the dHL-60 cells showed higher effector functions than those from the HL-60 cells. These results suggest that the EVs derived from the dHL-60 cells could be attractive vehicles for drug delivery.

In recent years, substantial effort has been undertaken to develop a drug-delivery system using cell-derived EVs. Although extensive studies, including clinical trials, are ongoing to develop drug-delivering EVs, several limitations still remain. Therefore, recent studies have searched for an alternative source of drug-delivering EVs and immune cells are strong candidates^11,12^. Immune cells actively release EVs upon external stimulation, especially at inflammatory sites. Immune cell-derived EVs are equipped with multiple receptors and ligands derived from their parent cells and contain multiple effector molecules such as miRNAs. Therefore, they are considered to be an appropriate drug-delivering EVs without possible toxic reactions^11,15^. In the current manuscript, we suggest that the neutrophil-like dHL-60 cells could be another candidate for the source of drug-delivering EVs. The dHL-60 cells generate massive amounts of EVs spontaneously (Fig. 1b), and the sizes of EVs are large enough to load various cargoes (Fig. 1c). Although the phenotype of the dHL-60-derived EVs is similar to NDEVs, they lack the effector function of NDEVs, such as bactericidal activity (Supplementary Fig. 3a) and chemoattractive effects on monocytes (Supplementary Fig. 3b).

Another issue to be solved in developing a drug-delivery system using EVs is cargo-loading techniques. The most common method is passive cargo loading by incubating small cargoes with isolated EVs. However, this method is time-consuming and has a low loading efficiency. Moreover, some cargoes adhere to surfaces of the EVs rather than being adequately incorporated. Therefore, active loading techniques are employed to increase the efficiency of cargo loading. The cargoes were loaded using electroporation or membrane disruption (e.g., sonication, osmotic shock, freeze–thaw, and membrane permeabilization) and these techniques guaranteed high drug-loading efficiency. However, these techniques lead to disturbances in the membrane integrity of EVs and extensive molecule aggregates^1,8^, leading to decreased loading efficiency^13,41–44^. Therefore, the endogenous loading method—isolation of the cargo-loaded EVs from the parent cells passively incubated with cargoes—was developed, and this technique limits these drawbacks without compromised membrane integrity. However, low cargo-loading efficiency in this method should be solved^42,44^.

We used well-established endogenous cargo loading into HL-60 cells using transfection rather than merely incubating cells with the desired cargo^44^. The HL-60 cells are efficiently transfected cells with known transfection techniques. Therefore, they are used to evaluate general transfection efficiency for transfection materials. We employed mild biphasic shocks in the HL-60 cells, which greatly enhanced the overall cargo-loading efficiency of small molecules (Figs. 2 and 3) and even large proteins (Fig. 4) to reduce possible membrane damage by transfection. The aggregation and disruption of EVs were significantly reduced because possible harmful steps, such as freeze/thaw and sonication, were missing in our method. In addition, we found that the dHL-60 cells generated higher amounts of EVs spontaneously within a given period. The dHL-60 cells showed higher incorporation of desired cargoes, and the dHL-60-derived EVs showed higher incorporation with higher cargo efficiency (Figs. 2, 3, 4). These results suggest the advantages of EVs derived from the HL-60 and dHL-60 cells as drug-delivery vehicles. Moreover, the dHL-60 cells generate tremendous amounts of EVs within minutes, and it is an essential advantage for developing drug-delivery vehicles.

In conclusion, we provided evidence that neutrophil-like dHL-60 cells quickly produce a tremendous amount of EVs. We also demonstrated that endogenous loading of the dHL-60 cells by electroporation could attribute desired functionality into isolated EVs. Therefore, our study highlights the increased productivity and efficient loading of therapeutics into dHL-60 cell-derived EVs. This finding opens the possibility for generating a drug-delivery system using immune cells, especially neutrophils.

## Supplementary Information

Supplementary Information

## Abbreviations

EV: Extracellular vesicles
NDEV: Neutrophil-derived extracellular vesicles
HL-60: Human promyelocytic leukemia cells
dHL-60: Neutrophil-like differentiated human promyelocytic leukemia cells
ATRA: All-trans-retinoic acid
fMLP: N-formyl-methionyl-leucyl-phenylalanine
MCP-1: Monocyte chemoattractant protein 1
iNOS: Inducible nitric oxide synthase
