# Supplementary material for: Extracellular vesicles from dHL-60 cells as delivery vehicles for diverse therapeutics
Source: Sci Rep. 2021 Apr 15;11:8289. doi: 10.1038/s41598-021-87891-8 (PMC8050327; doi:10.1038/s41598-021-87891-8)
Supplement: Supplementary file 1 — Supplementary Information [file 41598_2021_87891_MOESM1_ESM.docx]

**Supplementary Information**

**Development of drug-delivery system using dHL-60-derived extracellular vesicles**

Jun-Kyu Kim, Young-Jin Youn, Yu-Bin Lee, Sun-Hwa Kim, Dong-Keun Song, Minsang Shin, Hee Kyung Jin, Jae-sung Bae, Sanjeeb Shrestha, Chang-Won Hong

**Supplementary Methods**

**Supplementary Figure Legends**

**Supplementary Figures 1-4**

**Supplementary Methods**

**Morphology assessment of dHL-60 cells.** Vehicle-treated and ATRA-treated HL-60 cells were collected on Day 0, Day 3, Day 6, and Day 9. Cells were cytospunned for 5 min at 1500 rpm onto glass slides. The slides were stained with wright-giemsa stain (Merk Millipore) and images captured at 20 × objective (Nikon).

**The quantification of apoptosis of dHL-60 cells.** Vehicle-treated and ATRA-treated HL-60 cells were collected on Day 0, Day 3, Day 6, and Day 9. The cells were washed and resuspended in 200 μl of apoptosis staining solution (Abcam). The cells were stained with 5 μl of propidium iodide (PI, Abcam) and annexin V (Abcam). The cells were acquired using BD FACS Calibur and the percentage of apoptotic cells determined.

**Quantification of neutrophil extracellular trap formation from dHL-60 cells.** Naïve HL-60 cells or dHL-60 cells (5 × 10^5^ cells) were seeded and stimulated with 0-1000 ng/ml of PMA (Sigma-Aldrich) for 1 h at 37 ℃. Cells were then stained with 5 μM of sytox green (cell-impermeable DNA staining dye, Molecular probes) for 20 min. Then, cells were washed with × PBS, resuspended in 1 × PBS, and fluorescence levels were measured using spectrophotometer (Spectramax M2/e fluorescence microplate reader, Molecular devices).

**ROS generation by dHL-60 cells.** Naïve HL-60 cells or dHL-60cells (5 × 10^5^ cells) were seeded and stimulated with 0-1000 ng/ml of PMA (Sigma-Aldrich) and incubated for 1 h at 37 ℃. Then, cells were then stained with 5 μM of DCF-DA (Molecular probes) for 20 min. Cells were washed with 1 × PBS, resuspended in 1 × PBS and, the fluorescence levels of oxidized DCF were measured using spectrophotometer (Molecular devices).

**Degranulation in dHL-60 cells.** Naïve HL-60 cells or dHL-60 cells ( 1 × 10^6^ cells) were stimulated with 1μg/ml of PMA (Sigma-Aldrich). Cells were fixed and stained with CD63 (FITC, BD bioscience), CD66b (FITC, BD bioscience) and CD35 (PE, BD bioscience) for 1 h. Then cells were washed and resuspended in stain buffer. The fluorescence-labelled cells were acquired using flow cytometer and the expression levels of each degranulation markers were analyzed using flowjo (Treestar Inc).

**qPCR analysis.** RNA was extracted from THP-1 cells exposed to EVs using TRIzol (Ambion) and cDNA was synthesized using RT^2^ First strand kit according to manufacturer’s recommended protocols (Qiagen). RT-qPCR was performed by using cDNA (100 ng), primer (1 μl) and RT^2^ SYBR Green qPCR mastermix (Qiagen) using Qiagen rotor. Pre-designed RT^2^ qPCR primer assays (Qiagen) were used to determine the expression level of miR-16

**Supplementary Figure Legends**

**Supplementary Figure 1. Morphology and apoptotic rates of HL-60 cells treated with ATRA.** (**a**) Naïve HL-60 cells were treated with ATRA 1uM for 9 days and the morphology was determined by hemacolor staining in Day 3, Day 6 and Day 9. (**b**) Apoptosis rate of ATRA treated HL-60 cells were determined by FACS analysis of Annexin V and propidium iodide stained cells.

**Supplementary Figure 2. Surface marker expressions and effector functions of HL-60 cells treated with ATRA.** (**a**) Surface marker expression of HL-60 cells and dHL-60 cells on Day 3, Day 6 and Day 9. (**b, c**) NETs release and ROS generation by naïve HL-60 cells and dHL-60 cells treated with different concentration of PMA. (**d**) Degranulation markers in naïve HL-60 cells and dHL-60 cells after treatment with PMA.

**Supplementary Figure 3. Function of naïve HL-60 cells and dHL-60 cells derived MVs.** (**a**) Bactericidal activity of HL-60 cells and dHL-60 cells derived EVs against *E. coli* and *S. aureus*. (**b**) Monocyte chemotaxis towards HL-60 cells and dHL-60 cells derived EVs.

**Supplementary Figure 4. Incorporation of cargo in HL-60 cells and dHL-60 cells derived EVs.** (**a**) Dot plot showing transfection efficacy of indicated cargo into naïve HL-60 cells or dHL-60 cells. (**b**) Dot plot showing expression of transfected cargo in EVs derived from naïve HL-60 cells and dHL-60 cells. (**c**) Dot plot showing uptake of Cas9 loaded EVs derived from naïve HL-60 cells and dHL-60 by dTHP1, MCF-7 and Jukart cells. (**d**) C(t) value of miR-16 in dTHP-1 cells exposed to EVs derived from HL-60 cells and dHL-60 cells.

**Supplementary Figure 1**

**Supplementary Figure 2**

**Supplementary Figure 3**

**Supplementary Figure 4**
